# Dissecting regulatory T cell expansion using polymer microparticles presenting defined ratios of self-antigen and regulatory cues

**DOI:** 10.3389/fbioe.2023.1184938

**Published:** 2023-06-27

**Authors:** Christopher J. Bridgeman, Shrey A. Shah, Robert S. Oakes, Christopher M. Jewell

**Affiliations:** ^1^ Fischell Department of Bioengineering, University of Maryland College Park, Baltimore, MD, United states; ^2^ United States Department of Veterans Affairs, Baltimore, MD, United states; ^3^ Robert E Fischell Institute of Biomedical Devices, University of Maryland College Park, Baltimore, MD, United states; ^4^ Department of Microbiology and Immunology, University of Maryland Medical School, Baltimore, MD, United states; ^5^ Marlene and Stewart Greenebaum Cancer Center, Baltimore, MD, United states

**Keywords:** microparticle and nanoparticle, autoimmunity, mTOR, antigen-specificity, tolerance, biomaterial, vaccine, immunotherapy

## Abstract

Biomaterials allow for the precision control over the combination and release of cargo needed to engineer cell outcomes. These capabilities are particularly attractive as new candidate therapies to treat autoimmune diseases, conditions where dysfunctional immune cells create pathogenic tissue environments during attack of self-molecules termed self-antigens. Here we extend past studies showing combinations of a small molecule immunomodulator co-delivered with self-antigen induces antigen-specific regulatory T cells. In particular, we sought to elucidate how different ratios of these components loaded in degradable polymer particles shape the antigen presenting cell (APC) -T cell interactions that drive differentiation of T cells toward either inflammatory or regulatory phenotypes. Using rapamycin (rapa) as a modulatory cue and myelin self-peptide (myelin oligodendrocyte glycoprotein- MOG) – self-antigen attacked during multiple sclerosis (MS), we integrate these components into polymer particles over a range of ratios and concentrations without altering the physicochemical properties of the particles. Using primary cell co-cultures, we show that while all ratios of rapa:MOG significantly decreased expression of co-stimulation molecules on dendritic cells (DCs), these levels were insensitive to the specific ratio. During co-culture with primary T cell receptor transgenic T cells, we demonstrate that the ratio of rapa:MOG controls the expansion and differentiation of these cells. In particular, at shorter time points, higher ratios induce regulatory T cells most efficiently, while at longer time points the processes are not sensitive to the specific ratio. We also found corresponding changes in gene expression and inflammatory cytokine secretion during these times. The *in vitro* results in this study contribute to *in vitro* regulatory T cell expansion techniques, as well as provide insight into future studies to explore other modulatory effects of rapa such as induction of maintenance or survival cues.

## Introduction

Multiple sclerosis (MS) is an autoimmune disease whereby dysfunctional immune cells attack myelin that insulates neurons in the central nervous system leading to loss of motor control. In MS, innate and adaptive immune cells drive a proinflammatory state that leads to degradation of the myelin protein matrix (MPM) (Dendrou, Fugger and Friese, 2015). Although the pathogenesis of MS is incompletely understood, both the innate and the adaptive immune cell compartments contribute to disease progression. For example, antigen-specific B cells contribute to disease through the generation of myelin recognizing auto-antibodies (Arneth, 2019). Antigen presenting cells (APCs) such as dendritic cells (DCs) also initiate auto-activation of self-reactive T cells through the presentation of MPM-derived peptide antigen to myelin-specific CD4^+^ and CD8^+^ T cells (Gandhi, Laroni and Weiner, 2010; Weissert, 2013; Saxena et al., 2019). Once activated, these T cells drive disease through inflammatory cytokine production and targeted killing of host tissue expressing myelin antigens. Although therapies exist for MS, these require lifelong treatment and none are curative (Huang, Chen and Zhang, 2017). Further, even the most advanced therapies – such as monoclonal antibodies – do not distinguish between healthy and dysfunctional immune cells (Torkildsen, Myhr and Bø, 2016; Hauser and Cree, 2020). Thus, there is a need for new treatments that selectively control autoreactive immune cells while leaving the remainder of the immune system intact. Regulatory CD4^+^ T cells (T_REG_) are one target of particular interest that attenuate unwanted inflammation through the release of anti-inflammatory cytokines in response to self-antigens (Danikowski, Jayaraman and Prabhakar, 2017; Kimura, 2020). Indeed, *ex vivo* expanded T_REG_ have shown promise in clinical trials for treatment of autoimmune diseases such as type 1 diabetes and Crohn’s disease (Mosanya and Isaacs, 2019; Romano et al., 2019). However, these T_REG_ are polyclonal, and not necessarily specific to the disease-causing self-antigen. Alternatively, the expansion of disease specific T_REG_ may improve the potency and efficacy of these engineered cells. One emerging strategy to treat MS relies on the use of biomaterials to control the delivery of the T_REG_ promoting small molecule drug rapamycin (rapa) with MPM peptides to drive the expansion of antigen-specific T_REG_ that would protect against myelin degradation. This strategy fundamentally relies on the use of polymer microparticles (MPs) to control the release of these cues for myelin specific T_REG_ expansion *in vivo* (Tostanoski et al., 2016).

Polymer particle delivery strategies are an exciting area of research due to their ability to control drug delivery parameters, such as controlled release, drug co-localization, and tissue targeting (Andorko, Hess and Jewell, 2015; Tostanoski, 2016; Bookstaver et al., 2018). Poly(lactic-co-glycolic acid) (PLGA), for example, is a biodegradable polymer of particular interest due to its biocompatibility and degradation into non-toxic byproducts (Blasi, 2019; Su et al., 2021). Additionally, these particles are versatile in their ability to encapsulate a variety of immune cargos including proteins, peptides, and nucleic acids (Danhier et al., 2012). We and others have used poly(lactic-co-glycolide) (PLGA) microparticles loaded with myelin self-peptides, including myelin oligodendrocyte glycoprotein (MOG) and the mechanistic target of rapamycin (mTOR) inhibitor rapa to re-condition the microenvironment of lymph nodes (LNs) to promote antigen-specific T_REG_ (Tostanoski et al., 2016; Gammon et al., 2023). However, it is unclear how specific combinations and concentrations of these signals could further shape the response of APCs and T cells.

**FIGURE 1:**
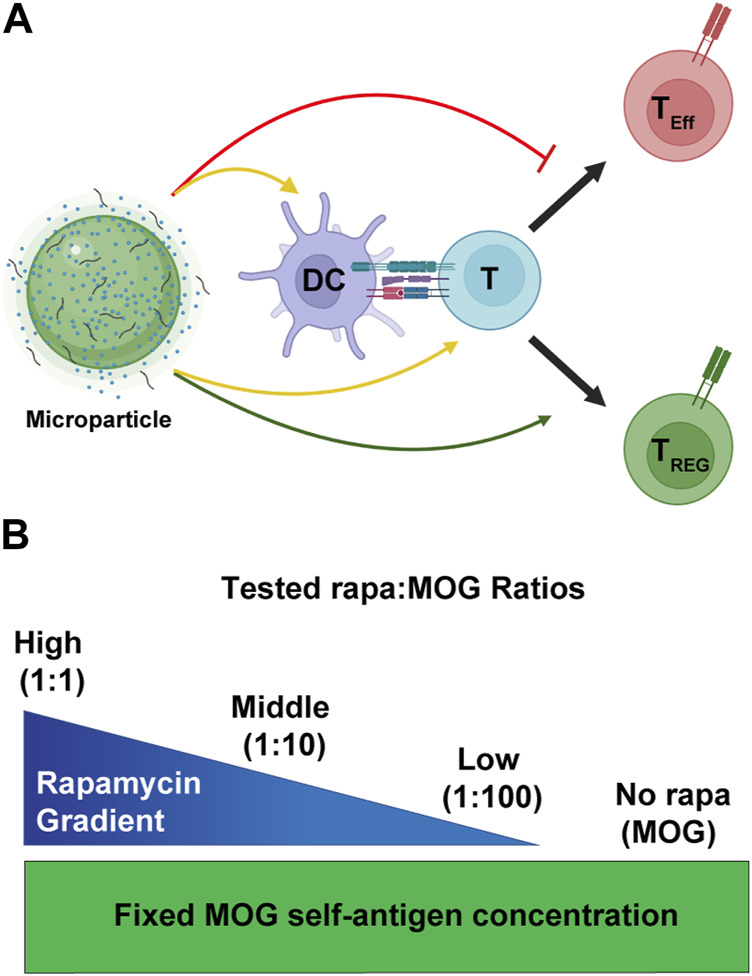
Overview schematic of MP treatment strategy. **(A)** MPs release immune cues that act on both DCs and T cells (yellow arrows). These cues modulate the interaction of DCs and T cells to promote generation of T_REG_ (green arrow) and not inflammatory effector T cells (T_Eff_). **(B)** To test the effect of rapa:MOG ratio, quantity of MOG peptide encapsulated in MPs was kept constant while rapa quantity was titrated. Created with BioRender.

The mTOR metabolic pathway has been shown to critically regulate cell functions such as proliferation, and metabolism (März et al., 2013; Mukhopadhyay et al., 2016; Yoo et al., 2017). While rapa has historically served a role of an important immunosuppressant (Saunders, Metcalfe and Nicholson, 2001; Yellen et al., 2011; Hester et al., 2012), over the last decade its ability to facilitate T_REG_ expansion and survival has been extensively explored (Marín Morales et al., 2019; Sato et al., 2021; Vakrakou et al., 2022). Additionally, rapa encapsulation in microparticle and nanoparticle systems for antigen-specific T_REG_ induction has been studied in multiple pre-clinical models of autoimmune diseases (Maldonado et al., 2015; Tostanoski, 2016; Gammon et al., 2023). However, more mechanistic studies are needed to deconvolute how specific ratios of encapsulated rapa:MOG fundamentally shape the APC-T cell signaling axis to promote antigen-specific T_REG_. This paper seeks to bridge this knowledge gap by using validated *in vitro* assays to test how specific ratios of rapa and MOG regulate APC activation, and subsequent antigen-specific T_REG_ polarization (Figure 1A). In particular, MPs were synthesized with specific ratios of rapa and MOG (Figure 1B) to test their effect on primary APCs and subsequent polarization of antigen-specific T cells to T_REG_. Using this library, we show all particles containing rapa reduced the activation of stimulated DCs, but these effects were insensitive to the ratio of rapa:MOG. Interestingly, however, the expansion of MOG-specific T cells could be maximized by tuning the rapa:MOG ratio. These results provide additional insight into design parameters for polarizing T cells toward T_REG_ for antigen-specific autoimmune therapies.

## Materials and methods

### Materials and reagents

MOG peptide (MOG_35-55_, MEVGWYRSPFSRVVHLYRNGK) was synthesized by Genscript (Piscataway, NJ, United States) with >98% purity. Rapamycin was purchased from LC Laboratories (Woburn, MA, United States). 50:50 poly(lactic-co-glycolide) (PLGA) was purchased from LACTEL Absorbable Polymers. High molecular weight poly(vinyl alcohol) (PVA) was purchased from Alpha Aesar (Tewksbury, MA, United States). Dichloromethane (DCM) was purchased from Sigma Aldrich (St. Louis, MO, United States). Dimethyl sulfoxide (DMSO), micro-bicinchoninic acid (mBCA) assay, and eBioscience FoxP3 Fixation/Permeation kit were purchased from Thermo Fisher Scientific (Waltham, MA, United States). Antibodies for flow cytometry, including BV605-CD11c, v450-CD25, PE-T-bet, PE-CD40, PE-Cy-7-CD4, PE-Cy-7-CD86, APC-FoxP3, APC-CD80 were purchased from BD Biosciences. Zombie NIR Fixable Viability Kit was purchased from BioLegend.

### Synthesis of microparticles

MPs were synthesized as previously described using a water-in-oil-in-water double emulsion technique (Tostanoski et al., 2016). Briefly, 500 µL of aqueous phase was prepared by dissolving 2.2 mg of MOG into water. An organic phase was prepared by first dissolving PLGA at 16 mg/mL in DCM for 1 h (PLGA-DCM). For rapa-loaded MPs, lyophilized rapa was dissolved in a PLGA-DCM aliquot to 0.22 mg/mL, and then titrated 1:10 in PLGA-DCM. Inner emulsion was formed by sonicating the aqueous phase with organic phase at 12 W for 30 s. This emulsion was then homogenized with 40 mL of 2% w/v PVA solution for 3 min at 16,000 RPM to form final emulsions. MP solutions were then stirred overnight to evaporate DCM. MPs were then poured through a 40 μm cell strainer, and centrifuged for 5 min at 5000g. After aspiration of supernatant, MPs were washed 3x with 1 mL of water (resuspend, spin 5 min 5000g). MPs were resuspended in a final volume of 1 mL of water.

### Microparticle characterization

MP size and distribution were calculated using dynamic laser scattering (DLS) (Horiba LA-950). Rapa and MOG loading were quantified by dissolving a known mass of dried MPs in DMSO. MOG concentration was determined using a mBCA assay according to the manufacturer’s instructions. Rapa concentration was determined by measuring ultraviolet/visible (UV/Vis) spectroscopy values and comparing to a standard curve of known concentrations. Loading efficiency was calculated by comparing measured quantities to initial loading quantities.

### Dendritic cell activation assay

All studies involving animals were carried out under the supervision of the University of Maryland Institutional Animal Care and Use Committee (IACUC) in compliance with local, state, and federal guidelines. To test the effect of rapa:MOG in MPs on DC activation, CD11c^+^ DCs were harvested from the spleens of female C57Bl/6J mice (Jackson Laboratories, United States) using a bead based CD11c positive selection kit according to manufacturer’s instructions (Miltenyi, United States). DCs were plated at 100,000 cells per well in a 96 well flat bottom plate in 100 µL of RPMI + L-Glutamine media supplemented with 10% fetal bovine serum, 55 µM β-mercaptoethanol, 1x non-essential amino acid, 10 mM HEPES, and 2 x Pen/Strep. Cells were then immediately treated with MP formulations matched to treat each well with 15 µg of MPs. MOG (no rapa) MPs, and empty MPs were used to control for the effect of MOG and MP effect respectively. To assess the effect of rapa on DC activation, cells were stimulated with 0.25 μg/mL lipopolysaccharide (LPS). DCs were cultured at 37°C for 24 h at 5% CO_2_. DCs were then sequentially Fc blocked for 20 min, washed, stained in NIR viability dye, and stained with anti-CD80, anti-CD86, and anti-CD40 antibodies for flow cytometry on a Beckman Coulter CytoFLEX. Median fluorescence intensity of markers was used to determine activation.

### T cell and dendritic cell co-culture assay

To test the effect of rapa:MOG on antigen-specific T cell proliferation, transgenic 2D2 mice that express a MOG specific T cell receptor (TCR) were used (Jackson Laboratories). DCs were first harvested as described, and treated with MP formulations. After 24 h, CD4^+^ T cells were isolated from 2D2 mouse spleens using a bead based negative selection kit (Stem Cell, United States). T cells were added in a 3:1 ratio to wells containing DCs (300,000 T cells per well). At 48 h post T cell addition, T cells were sequentially Fc blocked for 20 min, washed, and then stained in NIR viability dye for 20 min. Cells were then washed and stained with antibodies for anti-CD4 and anti-CD25. After 1 h, cells were washed and placed in fixation buffer. Cells were then intracellularly stained with antibodies for the expression of forkhead box P3 (FoxP3) and T-box 21 (T-bet) transcription factors. Cells were analyzed for the expression of these markers using a Beckman Coulter CytoFLEX.

### RT-qPCR analysis of gene expression

To test the effect of rapa:MOG MPs on CD4^+^ T cell gene expression, real time quantitative polymerase chain reaction (RT-qPCR) was used to look at the expression of pro-inflammatory and regulatory genes. DCs and CD4^+^ T cells were co-cultured as described. Twenty-four hours following T cell addition to DCs, the non-adherent T cells were removed, pelleted, and lysed using RNA lysis buffer (Zymo Research). After lysis, RNA isolation was performed followed by RNA quantification using a NanoDrop 2000c (Fisher Scientific). Complementary DNA (cDNA) amplification was then performed on 200 ng of RNA from each sample using the SuperScript IV VILO Master Mix kit and following the manufacturer’s instructions. cDNA samples were then stored at −80°C until RT-qPCR analysis. For RT-qPCR analysis, 1 µL of probe, 10 µL of Master Mix, 4 µL of cDNA, and 5 µL of molecular grade water were combined in a 96 well RT-qPCR reaction plate. T cell expression of interferon gamma (IFN-ɣ) was tested against control probes for glyceraldehyde 3-phosphate dehydrogenase (GAPDH) and beta-actin (ACTB). RT-qPCR and determination of cycle threshold (Ct) values were performed by a QuantStudio 7 Flex Real Time PCR System (Thermo Fisher Scientific). Delta Ct (ΔCt) was determined as previously described (Schmittgen and Livak, 2008). ΔCt values for cells treated with rapa:MOG were divided by the MOG (no rapa) ΔCt value to determine fold difference. Values are plotted as -log_2_(ΔCt).

### Statistical methods and analysis

Flow cytometry gating and analysis was performed in FlowJo (v 10.8.1). All statistical analyses were performed using GraphPad Prism (v 9.4.1). To test significant difference between two groups, an unpaired *t*-test was used. To test significance between multiple groups, an analysis of variance (ANOVA) was used followed by a Tukey post-hoc test for multiple comparisons. Significance was defined if *p* values were less than 0.05. Graphs were made using GraphPad Prism software.

## Results

### Defined ratios of rapa and MOG peptide can be predictably loaded into microparticles

**FIGURE 2:**
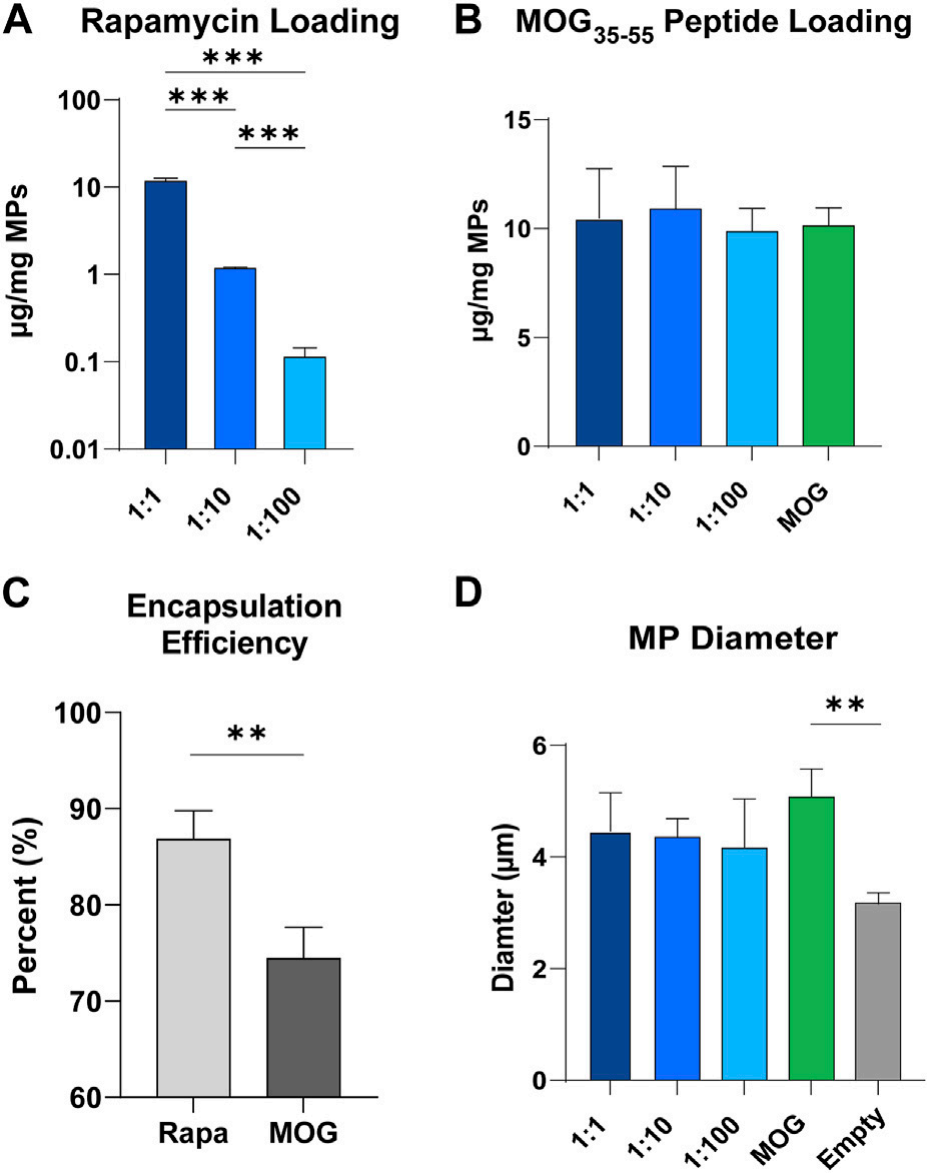
Characterization of MPs. **(A)** Quantity of rapa-loaded into MPs as measured through UV/Vis spectroscopy. N = 3 for each group. An ANOVA with a Tukey multiple comparisons post-hoc test was used to compare each group. Statistical significance is indicated by a (*). ***p < 0.001. **(B)** Quantity of MOG peptide loaded into MPs as measured through mBCA. **(C)** Loading efficiency of rapa and MOG into PLGA microparticles. An unpaired *t*-test was used to compare groups. Statistical significance is indicated by a (*). **p < 0.01. **(D)** MP size quantified through DLS. An ANOVA with a Tukey multiple comparisons post-hoc test was used to compare each group. Statistical significance is indicated by a (*). **p < 0.01.

An established double emulsion synthesis procedure was used to prepare particles with defined ratios of rapa:MOG (Figure 2A; Supplementary Figure S1) (Gammon et al., 2017; Gosselin, Tostanoski and Jewell, 2017). This process resulted in particles with direct control over the quantity of loaded rapa (Figure 2A) and peptide (Figure 2B). We observed a slightly larger standard deviation in the MOG loading into MPs co-loaded with high and middle quantities of rapa which could be attributed to charge interactions between hydrophobic rapa and PLGA, and hydrophilic MOG. This hypothesis is supported by our data showing that rapa had a significantly higher loading efficiency (87%) compared to MOG (74%) (Figure 2C). MP size was determined using dynamic laser scattering. These measurements revealed that MP diameter was not impacted by the specific rapa:MOG composition (Figure 2D). However, MPs compositions containing cargo were modestly larger than empty MPs. There was no significant difference between MPs loaded with rapa:MOG relative to MOG (no rapa) MPs; MP size was consistent with values previously reported from our lab (Tostanoski et al., 2016; Gosselin et al., 2021).

### Blunting of dendritic cell activation is not sensitive to the specific rapa:MOG ratio

**FIGURE 3:**
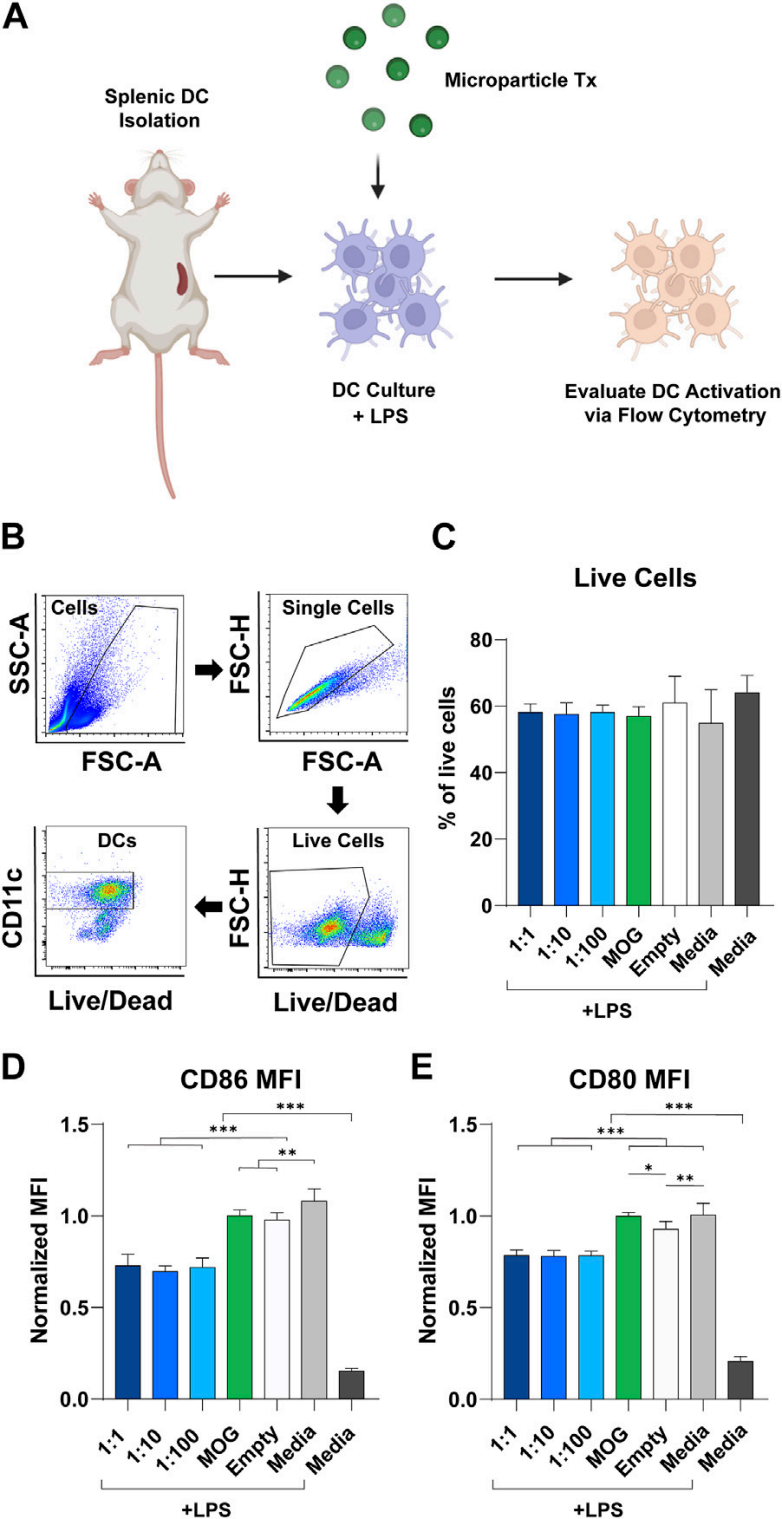
Effect of rapa:MOG MP treatment (Tx) on DC expression of activation receptors. **(A)** Overview schematic of experiment design. Primary derived DCs were harvested from mice and cultured with MP treatment for 24 h. DCs were then evaluated via flow cytometry for their expression of surface receptors. **(B)** Representative flow cytometry gating strategy. **(C)** Rapa:MOG MP treatment did not significantly reduce DC viability. **(D)** CD86 and **(E)** CD80 co-stimulation molecule expression on DCs was significantly lower compared to controls, but not within different rapa:MOG treatments. This study was completed in triplicate. For each study, N = 3. An ANOVA with a Tukey multiple comparisons post-hoc test was used to compare each group. Statistical significance is indicated by a (*). *p < 0.05, **p < 0.01, ***p < 0.001. Created with BioRender.

To study the modulatory effect of rapa:MOG ratio, primary derived wildtype splenic DCs were isolated, activated using LPS, then and treated with MPs (Figure 3A). After 24 h, DC expression of activation markers was characterized using flow cytometry. Live cell population was determined using a fixable viability stain, followed by positive selection of DCs based on their expression of CD11c (Figure 3B). Quantification of live cells showed that rapa:MOG MP treatment did not significantly impact cell viability compared to controls (Figure 3C). CD11c^+^ DCs were then analyzed for their expression of co-stimulation molecules CD86 (Figure 3D) and CD80 (Figure 3E) which play an important role in DC-T cell engagement. All groups treated with MPs containing both rapa and MOG exhibited reduced expression of CD86 and CD80 relative to control samples pulsed with LPS, including empty MPs. However, these reductions were equivalent irrespective to the specific rapa:MOG ratio in the MPs. This indicates rapa:MOG MP treatment modulates DC CD86 and CD80 signaling under highly inflammatory conditions, as opposed to complete attenuation of cell activity, and that this modulation is relative insensitive to ratio. As this assay was designed to test the effect of rapa:MOG ratio directly on DCs, we next wanted to test the effect of rapa:MOG treatment in the context of both DCs and T cells.

### Rapa:MOG microparticle treatments significantly increase T_REG_ frequencies 96 h after treatment

**FIGURE 4:**
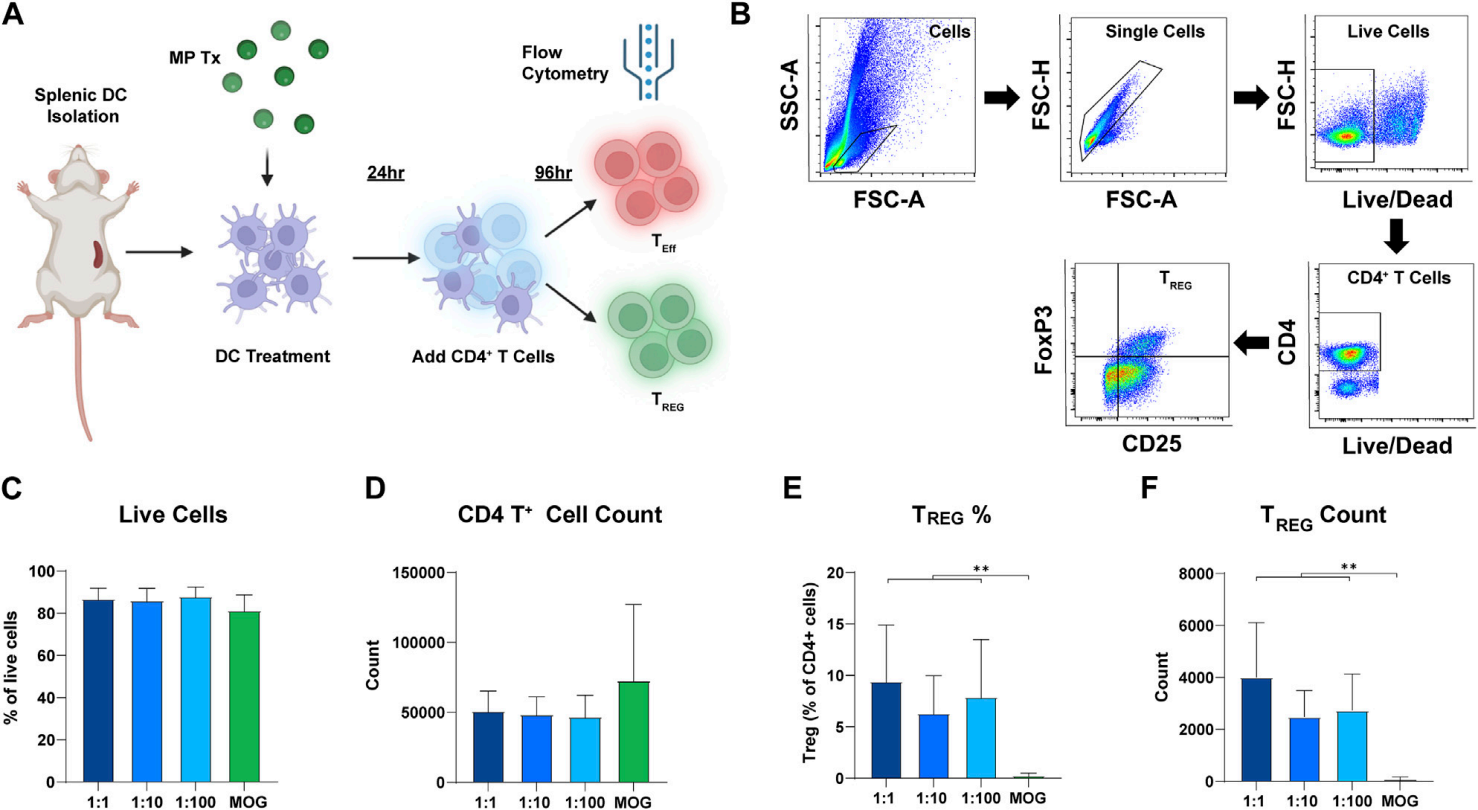
Results of DC-CD4 T cell co-culture experiment after 72 h. **(A)** Overview schematic of co-culture assay. **(B)** Representative gating strategy. **(C)** Viability was not significantly different between groups. **(D)** CD4^+^ T cell count was not significantly different between groups. **(E)** T_REG_ percentage of CD4^+^ T cells was significantly increased compared to MOG (no rapa) control. **(F)** Similarly, T_REG_ count confirmed that the number of T_REG_ was significantly higher compared to the MOG (no rapa) control. This study was completed in triplicate. For each study, N = 3. An ANOVA with a Tukey multiple comparisons post-hoc test was used to compare each group. Statistical significance is indicated by a (*). **p < 0.01. Created with BioRender.

Next, we tested if the ratio of rapa:MOG in MPs differentially expands antigen-specific T_REG_ *in vitro.* DCs were cultured with each MP formulation for 24 h. Transgenic CD4^+^ T cells expressing a MOG specific TCR were then isolated from 2D2 mice and co-cultured with the DCs (Figure 4A). After 96 h, cells were stained for viability and T cell phenotype markers to characterize their polarization as a function of ratio (Figure 4B). Increasing the ratio of rapa:MOG loaded into MPs did not decrease CD4^+^ T cell viability (Figure 4C). MOG (no rapa) MP treated cells on average had a higher trend in CD4^+^ T cell count compared to rapa:MOG treatments, but this was not significant (Figure 4D). This is likely due to the absence of the restraining effect of rapa in the control group, leading to more rapid T cell expansion. However, at this 96-hour expansion timepoint, polarization of T_REG_ (CD4^+^/CD25^+^/FoxP3^+^) percentage was not dependent on the specific ratio of rapa:MOG (Figure 4E). This result was also observed in our analysis of T_REG_ count confirming that rapa:MOG treatment increased polarization of T_REG_ independent of ratio (Figure 4F).

### Rapa:MOG promotes T_REG_ expansion and anti-inflammatory gene expression at 48 h post addition

**FIGURE 5:**
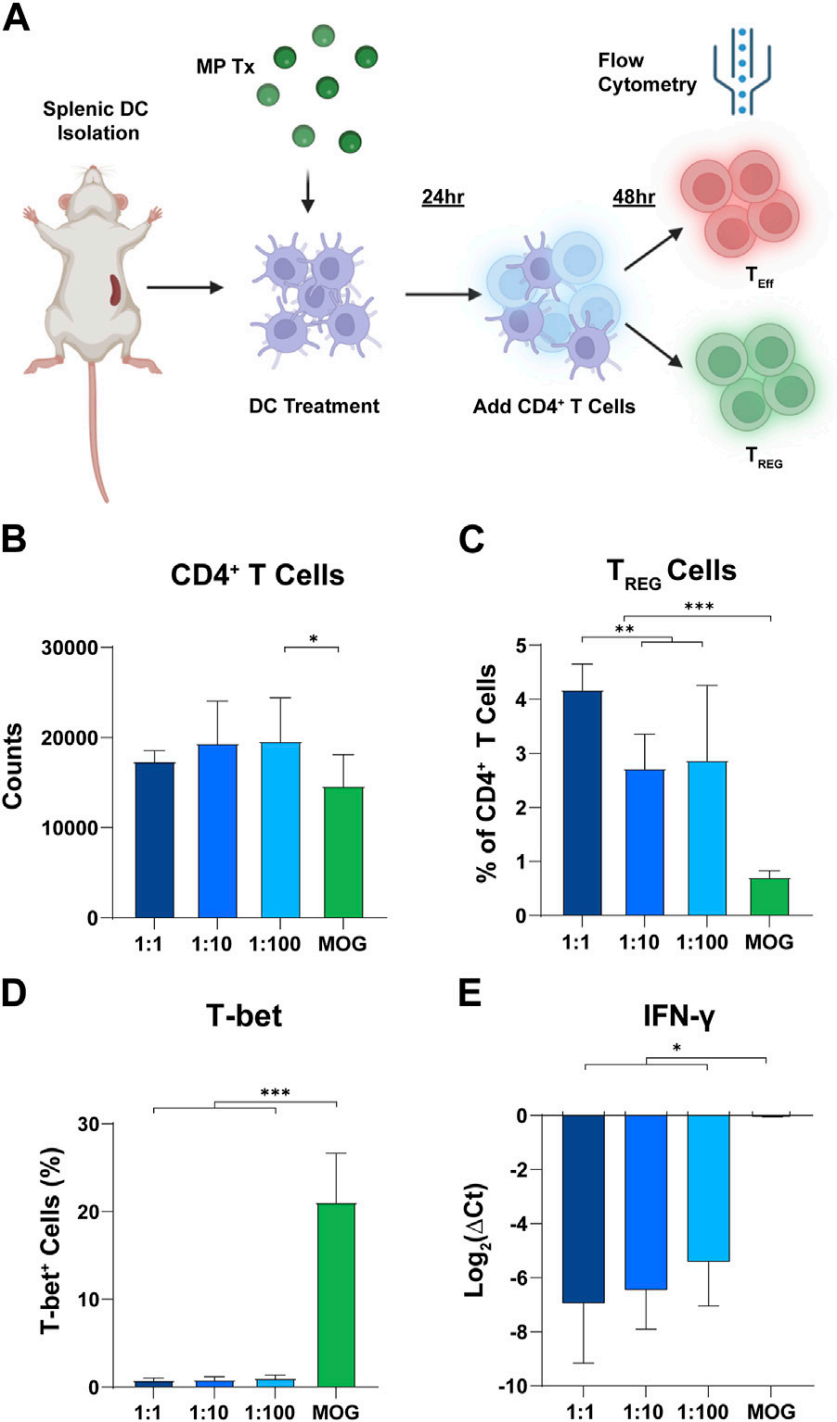
High rapa:MOG promotes greater expansion of T_REG_ after 48 h. **(A)** Overview schematic of co-culture assay. **(B)** There was no significant difference in T cell count between rapa:MOG treated groups. **(C)** High ratio of rapa:MOG induced the highest percentage of T_REG_. All groups induced significant increase in T_REG_ percentage over MOG (no rapa) control. **(D)** Rapa:MOG polarized cells away from expression of the inflammatory transcription factor T-bet. **(E)** RT-qPCR analysis showed rapa:MOG treated cells had significantly lower expression of the gene for the inflammatory cytokine IFN-ɣ. This study was completed in triplicate. An ANOVA with a Tukey multiple comparisons post-hoc test was used to compare each group. Statistical significance is indicated by a (*). *p < 0.05, **p < 0.01, ***p < 0.001. Created with BioRender.

Since T cell polarization and expansion is a dynamic process, we hypothesized that differences in rapa:MOG polarization may be distinguishable at earlier timepoints of T cell-APC engagement. To test this hypothesis, we analyzed T cells after 48 h of co-culture instead of 96 h (Figure 5A). Interestingly, we observed a significant increase in T cell counts of low rapa:MOG treated cells compared to MOG (no rapa) treated cells (Figure 5B). Similar to the 96-h timepoint, rapa:MOG MP treatment significantly expanded T_REG_ across all tested ratios. Excitingly, however, there was a significant dependence on the ratio of rapa:MOG for the polarization of T_REG_ percentage (Figure 5C). Since we observed this ratio dependent trend in T_REG_, we hypothesized that there would also be a decrease in functional inflammatory profile of T cells. First, we used intra-cellular antibody staining to assess the transcription T-bet which is associated with inflammatory T cell subtypes, and found that rapa:MOG treatment significantly reduced T-bet expression (Figure 5D). We also used RT-qPCR to assess T cell gene expression for a key functional cytokine produced during inflammation, IFN-ɣ, and found that the highest rapa:MOG treatment led to the lowest expression of the IFN-ɣ gene (Figure 5E). Taken together, these results indicate MPs loaded with a high ratio of rapa:MOG MP play an important role in polarizing antigen-specific T cell differentiation to T_REG_ and correspondingly decreasing expression of inflammatory markers.

## Discussion

Biomaterial enabled strategies for drug delivery are gaining increasing interest due to their ability to precisely control release parameters such as time, location, and concentration. Particularly, rapa delivery through both microparticle and nanoparticle formulations *in vivo* has shown significant immune engineering promise across multiple species models in the study of chronic inflammation, transplant rejection, and auto-immune disorders (Fan et al., 2019; Fisher et al., 2019; Nguyen et al., 2022). Although many biomaterial vehicles may be used to facilitate rapa delivery, much work continues to rely on the use of PLGA due to its versatility and extensive use in U.S. Food and Drug Administration (FDA) approved products (E. Emerson et al., 2020). More recently, the addition of specific self-antigens to rapa delivery strategies has shown promise in directing the formation of antigen-specific T_REG_. The work in this paper builds off of previous studies that have shown that co-encapsulation of self-antigen (MOG) and a modulatory cue (rapa) can promote the polarization of antigen-specific regulatory T cells for treatment of pre-clinical model of MS (experimental autoimmune encephalomyelitis) (Tostanoski et al., 2016). Specifically, this paper studies how the ratio of rapa and MOG co-localized in MPs controls the activation of APCs and the formation of T_REG_ *in vitro*.

Diffusion limited MPs rely on their size for retention within the LN in order to facilitate the direct release of cues to the tissue (Jewell, Bustamante López and Irvine, 2011). Although the quantity of rapa was decreased by over 100-fold, particles retained previously reported engineered diameter that would allow for future *in vivo* testing (Jewell, Bustamante López and Irvine, 2011). Additionally, particle size plays an important role in phagocytic uptake, which could have implications for *in vitro* T_REG_ expansion therapies. Interestingly, both the hydrophilic MOG cargo, and hydrophobic rapa cargo had an influence on MP size. However, our results showed that particles maintained a diameter that would permit phagocytosis.

APC co-stimulation is an important requirement for T cell activation and polarization into either an inflammatory or anti-inflammatory phenotype. However, rapa is a potent mTOR inhibitor that has been previously linked to reduced expression of co-stimulation receptors on APCs (Carey, Gammon and Jewell, 2021; Gosselin et al., 2021). Thus, it is important to understand if high ratios of rapa:MOG have a deleterious effect on DC viability and co-stimulation. Our study focused on the expression of the co-stimulatory molecules CD86 and CD80 which are the main ligands for the T cell activation receptor CD28 which is needed to promote T cell survival. The results of our experiments showed that DC expression of co-stimulation receptors was insensitive to rapa:MOG treatment. This suggests that both the low and high ratio of rapa:MOG exerted similar modulatory effects on DCs after 24 h.

To understand the effect of rapa:MOG ratio on the interaction of DCs and antigen-specific T cells, we co-cultured and treated primary derived DCs and primary derived transgenic MOG recognizing T cells. As the transgenic T cells only express one receptor, groups without MOG antigen had low viability and were excluded from analysis. Rapa is a well-known immunomodulator that drives proliferation of T_REG_, and our experiment showed that groups treated with MPs containing rapa indeed adopted a CD25^+^ FoxP3^+^ regulatory phenotype. Although the ratio of rapa:MOG did not significantly change the percentage of polarized T_REG_ after 96 h, we did observe significant ratio dependent changes after 48 h. The lack of significant difference between rapa:MOG treatments at later timepoints could potentially be attributed to the aggressive expansion kinetics of transgenic T cells engineered to recognize specific self-antigens. Alternatively, the half-life of rapa is approximately 60 h and may elicit its strongest effect at earlier timepoints. Additionally, T cells would not have undergone as many divisions at earlier timepoints, and thus the concentration of rapa per cell could have a more evident effect-potentially as a function of cell metabolism (Zhang et al., 2019). However, these ratio dependent differences were only observed for the percentage of T_REG_, and not for T-bet transcription factor expression or IFN-ɣ gene expression. Despite the highly constrained nature of this study, these results lay important groundwork for interrogating the role of rapa and self-antigen in directing anti-inflammatory responses at initial stages of treatment.

Although this study focused on testing how rapa:MOG ratios shape T_REG_ induction, it has been shown that rapa can have modulatory effects beyond T_REG_ induction such as in the generation of “memory” like phenotypes. Future work should seek to understand how the ratio of rapa and self-antigen modulate the adoption of memory cells which could be useful in engineering long lasting tolerizing T cell responses for treatment of auto-immune diseases. Although the concept of regulatory memory is continually evolving, metabolic reprogramming of T cells is known to play a critical role in cell fate. Interestingly, our results showed rapa:MOG led to a trend in lower T cell counts at 96 h, but actually resulted in a significant increase in the low ratio group at 48 h. This suggests that the MOG and rapa work synergistically to modulate cell functions such as metabolism and proliferation. Thus, future studies should attempt to mechanistically differentiate antigen-specific T_REG_ responses from non-antigen-specific responses which potentially carries important implications for *in vitro* T_REG_ expansion applications. Finally, the *in vitro* culture conditions assayed here do not fully recapitulate the complexity of the immune microenvironment. Thus, future studies should test how rapa:MOG ratios can shape efficacy or immune memory under *in vivo* conditions.

## Conclusion

Our work here showed that the ratio of rapa:MOG encapsulated in polymer MPs does not differentially alter the engineered properties of MPs for future *in vivo* intra-LN translation. Rapa:MOG ratio did not modulate APC expression of costimulatory receptors involved in T cell activation highlighting DC tolerance to rapa. Interestingly, the ratio of rapa:MOG differentially drives the expansion of T_REG_, and anti-inflammatory T cell profile of CD4^+^ T cells.

## Data Availability

The raw data supporting the conclusion of this article will be made available by the authors, without undue reservation.
