# Supplementary material for: Dissecting regulatory T cell expansion using polymer microparticles presenting defined ratios of self-antigen and regulatory cues
Source: Front Bioeng Biotechnol. 2023 Jun 27;11:1184938. doi: 10.3389/fbioe.2023.1184938 (PMC10334287; doi:10.3389/fbioe.2023.1184938)
Supplement: Supplementary file 1 [file DataSheet1.PDF]

Title: Dissecting regulatory T cell expansion using polymer microparticles presenting defined ratios of self-antigen and regulatory cues

Supplementary Information

Christopher J. Bridgeman[1], Shrey A. Shah[1], Robert S. Oakes[1,3], Christopher M. Jewell[1,2,3,4,5*]

[1] Fischell Department of Bioengineering, University of Maryland College Park

[2] Robert E Fischell Institute of Biomedical Devices, University of Maryland College Park

[3] United States Department of Veterans Affairs, Baltimore, Maryland

[4] Department of Microbiology and Immunology, University of Maryland Medical School

[5] Marlene and Stewart Greenebaum Cancer Center, Baltimore, Maryland

*** Correspondence:**

Christopher M. Jewell, cmjewell@umd.edu

Keywords: microparticle and nanoparticle, autoimmunity, mTOR, antigen-specificity, tolerance, biomaterial, vaccine, immunotherapy

This file includes:

Fig. S1

Table 1. PLGA MP Characterization for Rapa and MOG Signals

MP Formulation (Rapa:MOG)	Diameter (μm)	Cargo Loading (μg/mg MP)		Encapsulation Efficiency (EE) (%)	
		Rapa	MOG	Rapa	MOG
**1:1**	4.47 ± 0.39	12.48 ± 0.09	10.49 ± 1.31	89.88 ± 0.66	75.49 ± 9.43
**1:10**	4.35 ± 0.19	1.17 ± 0.02	10.90 ± 1.13	83.96 ± 1.79	78.45 ± 8.16
**1:100**	4.16 ± 0.51	0.12 ± 0.01	9.86 ± 0.61	86.59 ± 10.12	70.98 ± 4.41
**MOG**	5.07 ± 0.29	n/a	10.12 ± 0.48	n/a	72.88 ± 3.44
**Empty**	3.16 ± 0.11	n/a	n/a	n/a	n/a

*n/a indicates antigen or rapamycin was not present in the specific design
